# A 12‐Week Randomized, Double‐Blind, Placebo‐Controlled Trial for the Efficacy and Safety of a Novel Nutraceutical for Mild‐to‐Moderate Acne

**DOI:** 10.1111/jocd.70220

**Published:** 2025-05-05

**Authors:** Zoe Draelos, Julie Harper, Patricia K. Farris, Adina Hazan, Isabelle Raymond

**Affiliations:** ^1^ Dermatology Consulting Services, PLLC High Point North Carolina USA; ^2^ The Dermatology and Skin Care Center of Birmingham Birmingham Alabama USA; ^3^ Tulane University School of Medicine New Orleans Louisiana USA; ^4^ Nutraceutical Wellness Inc New York New York USA

**Keywords:** acne, acne vulgaris, nutraceutical, supplement

## Abstract

**Background:**

Acne is a prevalent skin concern that has many therapies targeting the pilosebaceous unit. Data suggest there are underlying immuno‐inflammatory drivers contributing to acne, with evidence for the efficacy of nutraceuticals including botanicals in targeting the key root causes. However, well‐designed clinical studies on their efficacy and safety remain scarce.

**Aims:**

The goal of this study is to determine the safety and efficacy of a novel oral nutraceutical in women with mild to moderate non‐nodulocystic acne.

**Methods:**

This 12‐week randomized, double‐blind, placebo‐controlled trial evaluated the safety and efficacy of a nutraceutical addressing mild‐to‐moderate acne in adult women. Subjects were washed out of all acne products and provided with a standardized skin regimen and randomized into active or placebo groups. The primary endpoint was the change in Investigator Global Assessment (IGA) of acne severity at week 12. Additional endpoints included improvements in inflammatory and non‐inflammatory lesion counts and blinded investigator skin assessments.

**Results:**

A total of 102 women were enrolled in the study, with 92 subjects completing the study (47 active; 45 placebo). Compared to placebo, subjects taking the supplement along with the same standardized skin care regimen saw significant improvements in IGA scores, and a higher percentage of them achieved clear/almost clear ratings. There were also significant decreases in both inflammatory and non‐inflammatory lesions compared to baseline, and overall skin parameters significantly improved compared to placebo.

**Conclusion:**

The oral nutraceutical significantly improves mild‐to‐moderate acne and overall skin health in adult women when compared to a placebo.

**Trial Registration:**

Clinicaltrials.gov identifier: NCT06097871

## 1 Introduction

Dermatologists have long considered acne vulgaris (AV) to be an inflammatory skin condition caused by a series of events that occur at the pilosebaceous unit (PSU) [1]. However, recent evidence suggests that there are also systemic drivers that play a role in the pathogenesis of acne [2]. These include stress, diet and metabolism, hormones, dysbiosis of the gut and skin microbiome, oxidative stress, and heightened immune responses [2]. These systemic drivers are interconnected and can trigger or exacerbate acne by upregulating immuno‐inflammatory pathways that ultimately affect the PSU [2]. Consequently, a system‐wide approach may be central in managing acne patients.

Traditional therapies for addressing AV at the PSU have focused on four factors: follicular hyperkeratosis, excess sebum production, loss of diversity in *Cutibacterium acnes* subtypes, and localized inflammation [1]. Treatment regimens targeting these factors include antibiotics (topical or oral), benzoyl peroxide, retinoids, salicylic acid, azelaic acid, and hormonal therapies including anti‐androgens and oral contraceptives [1]. These are commonly used in combination in order to multi‐target all four of the underlying mechanisms of AV at the PSU [3].

While there has been success in treating AV through directly targeting the PSU, emerging evidence on the role of systemic drivers of acne provides new therapeutic targets to be considered. Dietary changes, stress‐reducing techniques, and lifestyle modifications are becoming more mainstream recommendations for acne patients [4, 5]. A recent article by Burgess et al. [2, 6] reviewed in‐depth evidence supporting the use of botanicals, vitamins, and probiotics and postbiotics as part of a comprehensive approach to managing acne. For example, holy basil is a natural adaptogen used in ayurvedic medicine to address stress [7]. It is reported to decrease salivary cortisol levels and improve the physiological impacts of psychosocial stress [7, 8]. Plant‐based antioxidants could help mitigate oxidative stress and reduce lipid peroxidation and inflammation that contribute to acne [9, 10]. Berberine, a botanical traditionally used to improve glucose metabolism, has demonstrated beneficial effects in patients with acne and women with PCOS [11]. Mineral supplementation with zinc has proved effective for treating inflammatory lesions, and acne patients with low vitamin D levels improve with vitamin D supplementation [12, 13]. The role of probiotics and postbiotics is now being explored to improve or maintain a balanced microbiome, shown to be an integral part of acne resolution and skin health [14]. They have also been shown to augment the benefits and reduce side effects of minocycline when treating acne patients [15]. Maca, a Peruvian root that has been used to address female‐specific hormone fluctuations, was shown to increase estrogen and decrease luteinizing hormone (LH) and follicle‐stimulating hormone (FSH) in postmenopausal women [6, 16]. Again, this may be a useful approach to targeting some of the hormonal fluctuations that contribute to acne [16]. Finally, oral intake of konjac root extract, a root harvested for its ceramide content, has been associated with improving acne breakouts as well as an increase in hydration in the skin [17].

The ingredients shown in Table 1 as well as other key ingredients were carefully curated into a multi‐targeting oral formula. In a previous proof‐of‐concept study, both adult men and women taking this nutraceutical (Nutrafol SKIN) for 12 weeks saw significant improvements in their mild to severe acne as shown by improved IGA scores, lesion counts, and post‐inflammatory hyperpigmentation (PIH)/post‐inflammatory erythema (PIE) [2]. These results suggest that the nutraceutical addressing key root causes of acne shows promise in managing non‐nodulocystic acne [2, 27]. To more thoroughly investigate its skin benefits, a randomized, double‐blind, placebo‐controlled trial of this novel nutraceutical was conducted on adult females with non‐nodulocystic, mild to moderate acne.

**TABLE 1:** Root causes associated with acne and select ingredients in the novel nutraceutical.

Root cause associated with acne	Ingredient	Evidence
Stress	Holy Basil ( *Ocimum tenuiflorum* ) (Leaf)	An adaptogenic herb, clinically shown to reduce stress hormones [8, 18]
Diet & Metabolism	Berberine (*Berberis aristata*) (Root)	Supports healthy glucose metabolism [11]
Hormones	Maca HP (*Lepidium meyenii*, (Root) Selenium	Maca—An adaptogenic root, formulated to accompany selenium, which targets normal hormonal fluctuations [16, 19].
Gut & Skin Microbiome Dysbiosis	Probiotic ( *B. subtilis* DE111) Postbiotic ( *L. plantarum* L‐137)	Improves diversity of gut flora. Supports the breakdown and absorption of nutrients [20, 21]. Improves diversity of gut flora for healthy digestion and increases hydration in the skin [22].
Oxidative stress	Sicilian Orange ( *Citrus sinensis* ) Lycopene Olive Extract ( *Olea europaea* ) Ginger Extract ( *Zingiber officinale* , Rhizome)	Contains antioxidants to scavenge free radicals and support a healthy response to oxidative stress [10]. Antioxidant activity from tomatoes to support a healthy response to oxidative stress [23]. Harvested in Spain for its hydroxytyrosol content, known for its antioxidant properties [24]. Standardized for gingerols and extracted for its antioxidant activity [25].
Immune response	Curcumin	Promotes a healthy immune response and has antioxidant properties [26]

## 2 Materials and Methods

This was a 12‐week randomized, double‐blind, placebo‐controlled trial to test the safety and efficacy of an oral nutraceutical formulated to address mild to moderate acne in adult women. Study participants were healthy women aged 18–50 years old with mild‐to‐moderate acne. The study was approved by an Institutional Review Board (Allendale Investigational Review Board, Old Lyme, CT, USA), performed in accordance with the Declaration of Helsinki 1964 and its later amendments, complied with 21 CFR Part 56, and was conducted in compliance with good clinical practice. All participants provided informed, written consent prior to the initiation of any study‐related procedures.

### 2.1 Subject Selection

Subject eligibility was determined at a screening visit 1 week prior to baseline (BL). Subjects who were generally in good physical and mental health and met all of the selection criteria listed in Table 2 were included in the study. A washout protocol of acne treatments and/or therapies was implemented prior to the start of the study, with details found in Table 3.

**TABLE 2:** Subject selection criteria.

Inclusion criteria	Exclusion criteria
Women 18–50 years of age.	Women who are pregnant, lactating, or planning to become pregnant during the study or within 30 days of study completion.
Subjects with mild to moderate acne (5 inflammatory lesions, 10 non‐inflammatory lesions).	Subjects who have acne nodules/cysts representative of severe acne.
Subjects with an IGA score of 2–3	Subjects who are currently experiencing an acne flare.
Subjects with all skin types (normal, oily, etc.)	Any dermatological disorder, which in the investigator's opinion, may interfere with the accurate evaluation of the subject's skin characteristics, except for the study condition of acne.
Subjects with all Fitzpatrick skin types I–VI.	The subject has a history of or a concurrent health condition/situation that, according to the investigator, may put the individual at significant risk, confound the study results, or interfere significantly with the individual's participation in the study.
No known medical conditions that, in the investigator's opinion, may interfere with study participation.	The subject is taking medications that would mask an adverse event (AE) or influence the study results, including immunosuppressive drugs, steroidal and/or non‐steroidal anti‐inflammatory drugs within 3 months prior to the first visit, regular use of antihistamines, berberine, and/or those listed in Table 3.
	Subjects with any known allergies or sensitivities to the study products.

**TABLE 3:** Washout protocol. Time required from the previous use of products prior to the first visit.

Time	Products/Therapies
2 weeks	Light therapy Over‐the‐counter topical medications/products (including anti‐acne or antibacterial agents, topical anti‐inflammatories, topical retinoids, etc.). Sunscreens were acceptable.
1 month	Prescription (oral or topically applied on the face) antibiotics, inhaled steroids (except those prescribed for allergies). Hormones (insulin, etc.) Prescription oral medication for acne Topical prescription retinoids or other similar prescription drugs on the face.
3 months	Starting, stopping, or changing doses of hormone replacement therapy (HRT) or hormones for birth control (acceptable if the subject was on a stable dose for ≥ 3 months).
6 months	Isotretinoin or other oral retinoids

### 2.2 Study Procedures

A standardized skin care regimen consisting of a cleanser (Cetaphil Gentle Cleanser), moisturizer (Cetaphil Lotion), and sunscreen (Neutrogena Clear Face Sunscreen SPF30) was provided to all subjects at the screening visit. Subjects were instructed to use the provided products daily, beginning from the screening visit and throughout the course of the study. Subjects were instructed not to use any other skin products, undergo any professional or facial spa procedures, or introduce any new colored cosmetics on their face. They were also instructed to avoid sun exposure. Subjects were instructed to request topical rescue medication (0.1% Adapalene) if their acne worsened beyond what they considered personally acceptable.

One week after the screening visit and implementation of the standardized skin care regimen, subjects returned for the BL visit. At the BL visit, subjects were randomized using a computer randomization schedule to receive either the skin nutraceutical (SKIN, Nutrafol Skin Capsules, Nutraceutical Wellness Inc., New York, NY) or the placebo in a 1:1 active:placebo ratio. The placebo consisted of inactive ingredients, which included organic rice hulls, cellulose, artificial color, and brown rice flour. Both products were identical in physical characteristics and distributed in pre‐marked, de‐identified bottles. Subjects were provided with instructions to take four capsules of the investigational product once daily. The investigators and the subjects were kept blinded to the randomization throughout the course of the study. An unblinded coordinator did the product dispensing and had no other role in the study but to maintain the blind. All subject interactions were undertaken by a blinded coordinator and/or the blinded investigator.

Clinic visits occurred at BL, weeks 4, 8, and 12. Standardized photography was done at every visit using the Visia CR4.3 Photography System. Images of each subject were obtained for front, left, and right views of the face using both standard lighting and cross‐polarized lighting. Cross‐polarized lighting uses light filters to minimize glare and has been shown to best visualize skin qualities such as subsurface erythema, hyperpigmentation, hypopigmentation, subsurface vascularity, and inflammation [28].

The Investigator Global Assessment (IGA) was conducted in person at all visits using a 5‐point ordinal scale (0 clear, 1 almost clear, 2 mild, 3 moderate, 4 severe). The IGA is the FDA‐recommended scale to grade acne severity and assess treatment success in clinical studies [29]. The scores reflect both the quantity and quality of lesions on the face. A score of 0 or 1 was considered a successful treatment outcome, indicating clear or almost clear of acne. Inflammatory and non‐inflammatory lesion counts of the face were done at all study visits. The FDA considers lesion counts as a relevant and complementary assessment of product effect [29].

Investigator skin assessments were conducted at each clinic visit. PIE, PIH, acne scarring, overall skin tone, smoothness (visual), softness (tactile), and overall skin appearance were each rated on a 5‐point scale (0 = none, 1 = minimal, 2 = mild, 3 = moderate, 4 = severe). A rating of 0 or 1 was considered a positive outcome.

Instrumentation measurements were done at every clinic visit. Skin hydration was measured using a corneometer (Dermalab, Cortex Technologies, Denmark). Skin sebum was measured using a sebumeter (Courage Khazaka, Germany).

A self‐perception questionnaire developed by the sponsor was administered at visits 4, 8, and 12. The questionnaire consisted of 25 questions evaluating improvements in skin parameters compared to BL. These parameters included skin feeling hydrated, more even skin tone, fewer breakouts, acne visibly improved, and skin being clearer. Subjects rated changes compared to BL on a 4‐point or 5‐point scale (1 = strongly disagree, 2 = disagree, 3 = agree, 4 = strongly agree, not applicable where appropriate).

Daily diaries and pill counts were checked at clinic visits to ensure subject compliance. Adverse events (AEs) were documented and compiled throughout the duration of the study starting at the screening visit.

### 2.3 Study Endpoints

The primary outcome measurement was the change in IGA at 12 weeks in the active group using the standardized skin regimen compared to the placebo group using the standardized skin regimen, which henceforth will be simply described as active group vs. placebo.

Secondary endpoints were the improvement in investigator‐assessed IGA scores from BL, improvement (decrease) in lesion counts at 12 weeks for both inflammatory and non‐inflammatory lesions, improvement in investigator‐assessed skin parameters, improvement in corneometry and sebumeter measurements at all timepoints, and subject‐assessed improvement in skin parameters, all compared to BL and between active and placebo.

### 2.4 Statistical Analysis

Along with descriptive statistics (means, standard deviations, and percentages), ordinal non‐parametric data were analyzed using the Wilcoxon signed rank test, continuous data were analyzed using parametric tests such as a Student t‐test and ANOVA with post hoc Tukey HSD Analysis, and categorical data were analyzed using non‐parametric tests such as the chi‐square test and the binomial test of proportions. Change was considered significant at a *p* value less than or equal to 0.05 (*p* ≤ 0.05).

## 3 Results

A total of 102 women enrolled, with nine subjects who discontinued during the washout phase without having consumed any of the test materials or after BL assessments for personal reasons. The intent‐to‐treat population included 93 women, with 92 women completing the 12‐week study per protocol. One subject withdrew after consuming the active supplement and experiencing nausea deemed moderate in nature and possibly related to the study product.

A total of 47 women in the active group (mean age 30.8, range 18–49) and 45 in the placebo group (mean age 31.6, range 18–48) completed the study per protocol. Demographics were diverse in race and complexion type (oily, dry, combination, or normal) (Table 4), with no significant differences in subject demographics found between the active and placebo groups in age, race, complexion, or Fitzpatrick skin type (*p* > 0.05 for all parameters).

**TABLE 4:** Per Protocol subject demographics.

	Active (*n* = 47)	Placebo (*n* = 45)
Age (years)		
Mean (±SD)	30.8 (± 9.4)	31.6 (± 9.4)
Range (min., max.)	18–49	18–48
Race (*n*/%)		
African American	21 (45%)	20 (44%)
Caucasian	21 (45%)	20 (44%)
Hispanic	5 (10%)	5 (12%)
Complexion (*n*/%)		
Combination	26 (55%)	32 (71%)
Oily	12 (26%)	9 (20%)
Dry	8 (17%)	4 (9%)
Normal	1 (2%)	—
Fitzpatrick Skin Type (*n*/%)		
I	7 (15%)	6 (13%)
II	15 (32%)	14 (31%)
III	3 (6%)	5 (11%)
IV	4 (9%)	3 (7%)
V	12 (26%)	12 (27%)
VI	6 (12%)	5 (11%)

No subjects in the active or placebo group requested rescue medication (0.1% Adapalene) during study participation; therefore, no rescue medication was administered.

### 3.1 Primary Endpoint

**FIGURE 1:**
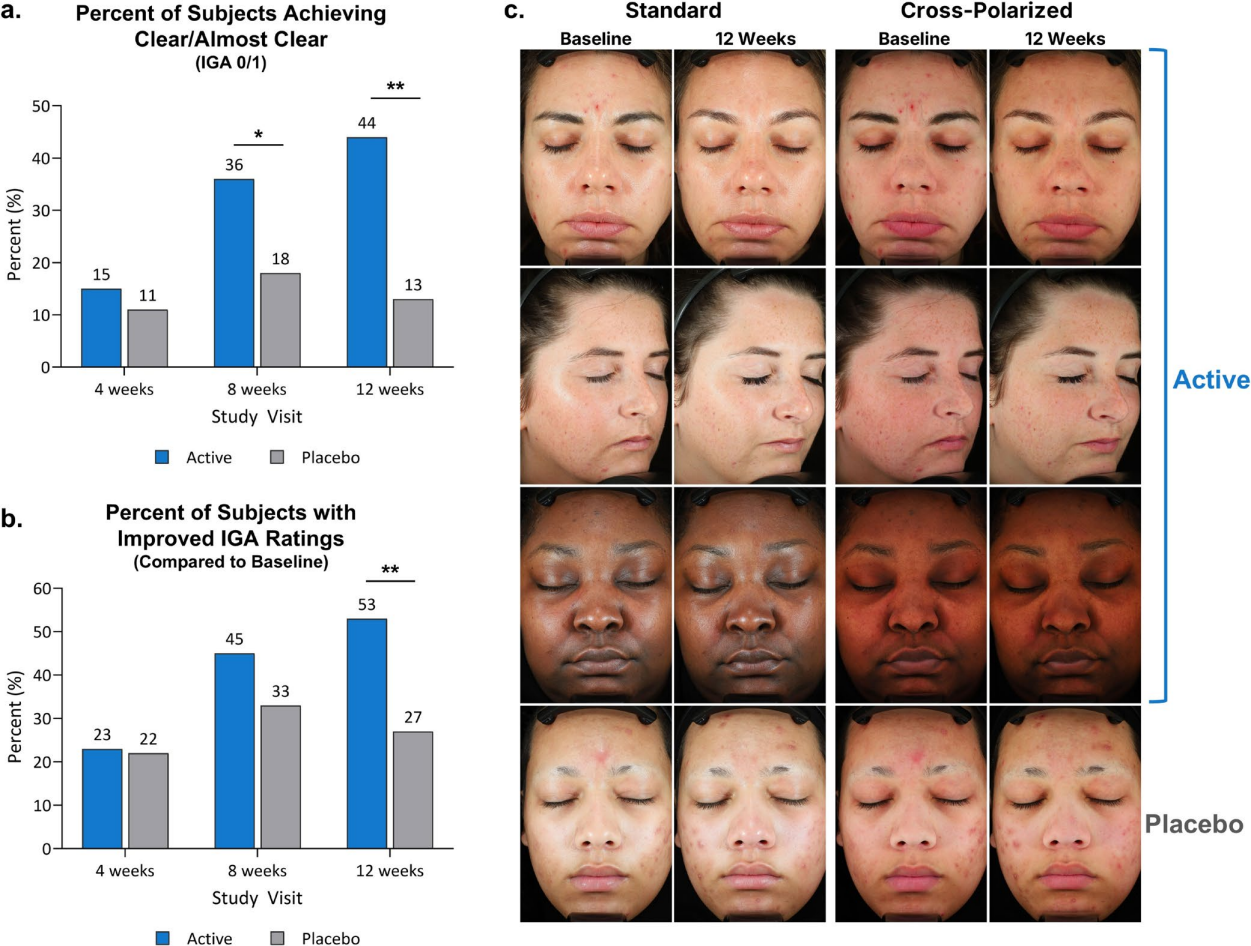
Improvements in IGA and acne severity in active versus placebo. (A) A significantly larger proportion of active subjects achieved ratings of clear or almost clear (IGA of 0 or 1) at weeks 8 and 12 compared to placebo subjects. (B) A significantly larger proportion of active subjects had improved IGA ratings at week 12 compared to placebo subjects. *p < 0.05 **p < 0.01 (C) Representative images of subjects using standard and cross‐polarized light in the active and placebo groups.

Average IGA ratings at BL were similar between the active and placebo groups (2.21, 2.33, respectively, *p* = 0.2), with all subjects beginning at ratings of 2 or 3 per the selection criteria. At week 12, the mean IGA score for the active group had significantly decreased to 1.47 (−0.74) compared to placebo at 1.98 (−0.36), achieving the primary endpoint (*p* = 0.02). The percentage of subjects achieving clear/almost clear (IGA rating of 0/1) was significantly greater in the active group by week 8 (36% active, 18% placebo, *p* < 0.05) and continued to increase to 44% in the active group compared to 13% in the placebo group by week 12 (*p* < 0.01, Figure 1a). The percentage of subjects improving over the course of the study was also significantly greater in the active group compared to the placebo group at week 12 (*p* < 0.01, Figure 1b). Representative subject images using standard and cross‐polarized light to visualize skin characteristics, as well as subsurface skin features, are shown in Figure 1c.

### 3.2 Secondary Endpoints

**FIGURE 2:**
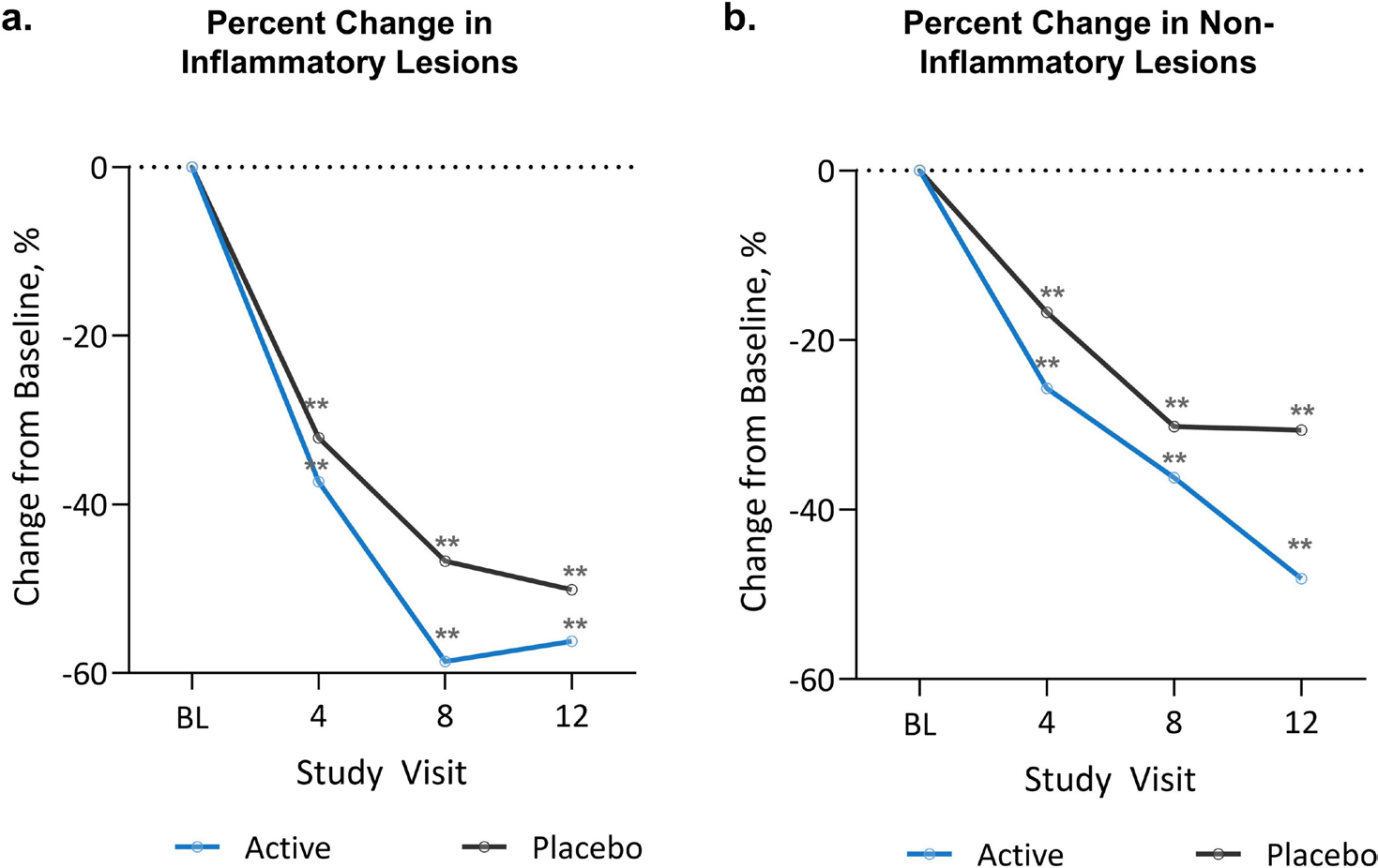
Decrease in lesion counts across study progression. (A) Inflammatory and (B) non‐inflammatory lesion counts significantly decreased over time in both the active and placebo groups. **p < 0.01 compared to BL.

Mean inflammatory lesion count at BL was 9.8 in the active group and 11.4 in the placebo group (*p* < 0.05). The observed mean changes from BL in active and placebo were −5.4 and −5.5, respectively. Due to the statistical difference in the starting BL values between the randomized groups, a covariate analysis was used to account for the impact that this difference had on the outcome measure. The adjusted mean changes from BL were −5.7 in the active group and −5.2 in the placebo group (*p* = 0.56). When compared to BL, inflammatory lesions in the active group decreased by 56.2% at week 12 (*p* < 0.01, Figure 2a). This was not statistically different compared to placebo; however, the mean percent decrease in inflammatory lesions trended larger in the active group than in the placebo group at each study time point (*p* > 0.05, Figure 2a).

At BL, the non‐inflammatory lesion count was 13.6 in the active group and 14.2 in the placebo group (*p* = 0.59). By week 12, the active group had a 48.1% decrease in non‐inflammatory lesions down to 7.3, which was significant compared to BL (*p* < 0.01). Although the percent decrease over time tended to be greater in the active group across the study duration, the difference between groups did not reach statistical significance in the change in non‐inflammatory lesions at week 12 (*p* = 0.16, Figure 2b).

**FIGURE 3:**
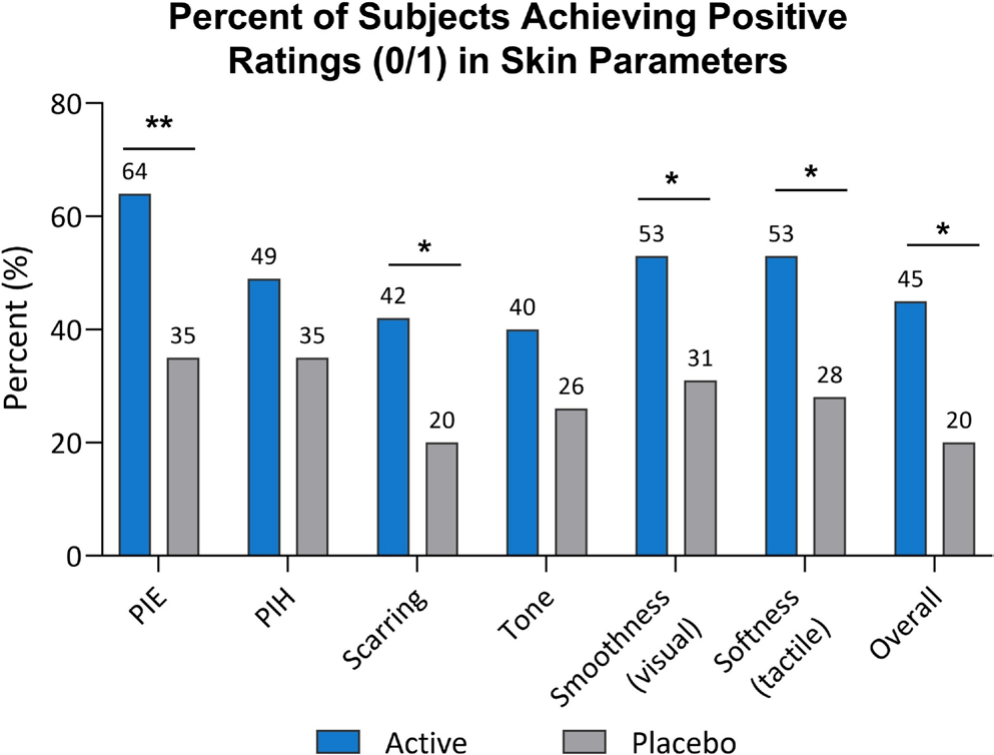
Improvements in skin parameters in active versus placebo. The active group had more subjects with positive ratings in all skin parameters at week 12. Significantly more subjects were rated as none or minimal (0 or 1) at week 12 in PIE, scarring, smoothness, softness, and overall skin improvement for the active versus placebo groups. *p < 0.05 **p < 0.01.

For the blinded investigator skin assessments, most subjects began with ratings of “2” or “3.” There were no statistical differences between groups in the percent of subjects with positive ratings (0/1) at BL. By week 12, a larger proportion of subjects in the active group compared to the placebo group achieved positive ratings for all skin parameter assessments, reaching significance for assessments of PIE (*p* < 0.01), acne scarring (*p* < 0.05), smoothness (*p* < 0.05), softness (*p* < 0.05), and overall skin appearance (*p* < 0.05, Figure 3).

Corneometer results were statistically unchanged throughout the study for either group. When looking at results over the course of the study, the active group measurements increased compared to BL (145.7) at all timepoints (4 weeks = 153.2, 8 weeks = 153.8, 12 weeks = 147.7), while results for placebo groups decreased compared to BL (153.3) at weeks 4 (149.7) and 8 (153.0) and increased at week 12 (159.1), though none of these changes reached statistical significance.

Sebum measurements throughout the study also remained statistically unchanged for both groups. BL measurements began at 69.3 in the active and 84.1 in the placebo (*p* > 0.05). Sebum measurements in both groups trended higher over the course of the study and ended with similar values (88.2 active vs. 97.0 placebo, *p* > 0.05).

In the subjective assessments, significant improvements were recorded in the active group at week 12 for improved skin tone (*p* = 0.02), visibly improved acne (*p* = 0.036), and clearer skin (*p* = 0.046) compared to BL. Subjects also reported healthier skin (*p* = 0.033) and that they would recommend the product (*p* = 0.011). However, these differences did not reach statistical significance compared to placebo.

### 3.3 Safety

The product was well tolerated. Of the 93 subjects in the intent‐to‐treat population, one subject in the active group reported experiencing nausea when consuming the supplement and subsequently withdrew from the study after BL. The AE was deemed moderate in nature and possibly related to the study product and resolved upon discontinuation. There were no other AEs documented throughout the study.

## 4 Discussion

The results of this randomized, double‐blind, placebo‐controlled study support the efficacy and safety of a novel oral supplement to improve mild to moderate, non‐nodulocystic acne in adult women. IGA scores were significantly improved over BL in the group taking the skin nutraceutical, and a higher percentage of them achieved clear/almost clear ratings. There were also significant decreases in both inflammatory and non‐inflammatory lesions compared to BL, and overall skin parameters significantly improved compared to placebo as rated by the blinded investigator.

While both IGA and lesion count are important for evaluating efficacy, the IGA is considered a more overall assessment of acne severity considering the overall impact of lesions, while lesion counts specifically measure the number of individual lesions reduction [29]. Though both inflammatory and non‐inflammatory lesion counts significantly improved in the active group compared to BL, the difference was not significant between groups at week 12. Nevertheless, the active group showed a greater reduction for both types of lesions at every time point. Furthermore, trends for the non‐inflammatory lesions also suggest a continuing decline in the active group, while the placebo group stabilized at week 12. Longer studies, perhaps of 16 or 20 weeks, should be considered in the future to evaluate the continued effects of the dietary supplement on outcomes.

Another potential reason for lesion counts not reaching statistical significance between groups at week 12 could be due to the BL starting point, which was defined as at least 5 inflammatory lesions and 10 non‐inflammatory lesions. In addition, because the nutraceutical targets key underlying drivers of acne instead of directly addressing the conventional PSU targets, it could be that the timelines for improvements are longer than those for more targeted conventional treatments. Additional studies on either more severe non‐nodulocystic acne or, as previously mentioned, longer studies may be useful to understand the extent of improvement in lesion counts under consistent use of the nutraceutical.

Although the supplement is designed to address key underlying drivers of acne, there were significant changes in skin parameters beyond acne. Significant improvements were reported in blinded investigator assessments for skin tone, PIE, scarring, smoothness, softness, and overall skin appearance for the active group compared to placebo. So in addition to improving acne, this nutraceutical improved the overall appearance of the skin, differentiating the oral supplement from other acne therapies, which are oftentimes associated with skin dryness, erythema, and skin irritation [30].

The placebo effect in acne studies is well documented. Studies show that there are and have been significant improvements in acne using both oral and topical placebo therapies [31, 32]. In addition, all subjects in the current study were placed on a standardized skin care regimen to reduce variability, but this could also increase the placebo effect and improve subjects' outcomes [33]. Therefore, it is not surprising that subjects in the placebo group showed improvements in many of the parameters measured [33]. Even with this, the group taking the oral supplement showed significant improvement over the placebo group in IGA, as well as blinded investigator skin‐assessed parameters. This data demonstrates that the oral supplement provides benefits for mild to moderate acne beyond a standardized skin regimen consisting of a cleanser, moisturizer, and sunscreen.

Reducing sebum production at the PSU has been a common target for therapies addressing AV. Even though AV severity decreased according to IGA and lesion counts, sebum levels throughout this study remained statistically unchanged over time and compared to placebo. Skin hydration (corneometer) was also statistically unchanged throughout the study. A previous study in men and women taking the same oral nutraceutical resulted in a decrease in sebum levels and an increase in skin hydration [2]. Further, the ingredient konjac root included in the nutraceutical has been shown to independently improve skin hydration [17]. The discrepancy seen in the current study may be explained by the fact that this study ran from spring to summer, which correlated with an increase in heat and humidity where the study was performed (North Carolina, USA). It has been demonstrated that heat and humidity do influence sebum production in the skin [34]. In addition, hydration measurements could have been affected by the standardized skin care regimen that the subjects were using [33]. Additional studies are needed to understand the effects of the oral supplement on sebum production and skin hydration.

In this study, only one AE of moderate nausea was reported and considered possibly related to the supplement. Otherwise, the supplement was well tolerated. Thus, the oral nutraceutical investigated in this study warrants consideration as an effective and safe approach to addressing mild to moderate acne.

In summary, the results of this study show that the well‐tolerated oral supplement significantly improves mild to moderate non‐nodulocystic acne as well as other parameters of skin appearance. In clinical practice, it is likely that this nutraceutical would be used in combination with other traditional therapies. Further studies exploring the effects of the oral supplement when taken in combination with conventional AV therapies would provide useful clinical insight.

## Author Contributions

Z.D. performed and supervised the assessments and data collection. J.H. and P.K.F. aided in interpreting the results. A.H. contributed to the writing of the manuscript. I.R. contributed to the design and implementation of the study and aided in the writing and editing of the manuscript. All authors discussed the results and provided input for the manuscript.

## Ethics Statement

All investigations were performed in accordance with the rules of the Declaration of Helsinki of 1975, under the approval of an Institutional Review Board (Allendale Investigational Review Board, Old Lyme, CT, protocol #DCS‐85‐23, 19 Oct 2023), comply with 21 CFR Part 56, and were conducted in compliance with good clinical practice. All participants provided written, informed consent before engaging in any study‐related procedures.

## Consent

A photo release form was obtained from all subjects prior to their participation in the study.

## Conflicts of Interest

Dr. Draelos received an educational grant from Nutraceutical Wellness Inc. to conduct the research detailed in this manuscript. Dr. Farris is an advisor for Nutraceutical Wellness Inc. Drs. Hazan and Raymond are employees of Nutraceutical Wellness Inc. Funding for this study was provided by Nutraceutical Wellness Inc.

## Data Availability

The data that support the findings of this study are available from the corresponding author upon reasonable request.
